# Inguinal hernias in patients on continuous ambulatory peritoneal dialysis: is tension-free mesh repair feasible?

**DOI:** 10.1186/s12893-020-00979-2

**Published:** 2020-12-02

**Authors:** Qiyu Chi, Zheng Shi, Zhibo Zhang, Chunzhong Lin, Guozhong Liu, Shangeng Weng

**Affiliations:** Department of Hepatopancreatobiliary Surgery, The First Affiliated Hospital of Fujian Medical University, and Fujian Provincial Institute of Abdominal Surgery, No.20 Chazhong Road, Fuzhou, 350005 Fujian China

**Keywords:** Inguinal hernia, Tension-free, Mesh repair, Peritoneal dialysis

## Abstract

**Background:**

Continuous ambulatory peritoneal dialysis (CAPD), which often causes a common complication such as abdominal wall hernia, is a prevalent alternative therapy for end-stage renal failure patients. However, relevant studies are somewhat rare, and the peritoneal dialysis (PD) protocol during the perioperative period is still controversial. The aim of this study was to evaluate the effectiveness and perioperative management of tension-free mesh repair for inguinal hernias in CAPD patients.

**Methods:**

Between January 2013 and December 2019, 18 CAPD patients with 20 inguinal hernias who underwent tension-free mesh repair were retrospectively analyzed. Data on demographics, perioperative features, the perioperative dialysis protocol and surgical complications were collected and assessed.

**Results:**

All hernias were diagnosed after the start of CAPD, and the median duration from PD onset to hernia formation was 16 months (2–61 months). All patients underwent successful tension-free mesh repair, including 17 Lichtenstein and 3 anterior Kugel procedures. The median operation time was 62.5 min, and the median postoperative hospital stay was 3 days. Fifteen patients received low-exchange volumes and high-frequency exchanges from 1 to 3 days after surgery for 2 weeks with gradual resumption of the original CAPD regimen within 4 weeks. Complications included seroma (n = 2) and hematoma (n = 1). No wound or mesh infection or recurrence occurred during the follow-up period.

**Conclusions:**

Tension-free mesh repair is safe and feasible for inguinal hernias in CAPD patients, The Lichtenstein mesh repair should be the first choice, and anterior Kugel repair may be considered an effective procedure. Bridging hemodialysis seems unnecessary except for emergency surgery.

## Background

Continuous ambulatory peritoneal dialysis (CAPD) is a prevalent and acceptable alternative therapy for patients with end-stage renal failure (ESRL) and has many economic benefits, is convenient, avoids the pain of establishing the vascular access needed for hemodialysis (HD) and provides a survival advantage while retaining residual renal function (RRF) [1–3]. Abdominal wall hernia is an unavoidable noninfectious complication in CAPD patients that is considered to be caused by the sustained increase in intra-abdominal pressure caused by injecting a large amount of dialysate during CAPD and by deterioration of abdominal wall tissue at this stage [4, 5]. The main types of hernias in CAPD patients include inguinal, umbilical, incisional and epigastric, and the former two types are the most common [4, 6].

Abdominal wall hernia is a major limiting factor of CAPD. When it develops, the effectiveness of CAPD becomes poor because dialysate can enter the scrotum through the abdominal wall defect and patent processus vaginalis and affect the efficiency of its ultrafiltration [7, 8]. Moreover, it may also cause patient discomfort, and even serious complications such as intestinal ischemia, necrosis and perforation. Given these reasons, hernioplasty in CAPD patients should be advocated. Only a few studies, including some case reports, have reported on hernioplasty of inguinal hernias in CAPD patients. Tension-free mesh repair for patients with CAPD has been reported to result in no recurrence [5, 9–11] or mesh infection [6]. However, these studies included a small sample size and need verification with more patients. In addition, the issues of perioperative management of bridging hemodialysis and time to restore the preoperative CAPD regimen are still under debate. The aim of this study was to evaluate the safety and effectiveness of tension-free mesh repair for inguinal hernias in CAPD patients.

## Methods

Between January 2014 and December 2019, 18 CAPD patients with 20 inguinal hernias who underwent tension-free mesh repair at the First Affiliated Hospital of Fujian Medical University were retrospectively analyzed. All surgeries are performed by a highly specialized hernia surgery team.

The preoperative examination included routine coagulation, spirometry, echocardiography, and regular ultrasonography and computed tomography (CT) to scan the herniated contents. Except for emergency surgery, all patients undergoing elective surgery received preoperative cardiopulmonary training. The patients were informed of the surgical regimen and perioperative management to obtain consent. CAPD was regularly performed until the day of surgery to ensure an optimal physical condition, and the dialysate was preoperatively drained from the peritoneal cavity. Cefmetazole, an intravenous prophylactic antibiotic, was administered in all of the patients and could be replaced by aztreonam if allergic reactions to penicillin or cephalosporins emerged.

An anesthesiologist was consulted preoperatively to develop the optimal anesthesia regimen. The basic principles of our institute are that most CAPD patients receive general anesthesia to improve their satisfaction and that local anesthesia is used only when general anesthesia and spinal anesthesia are contraindicated in patients with severe cardiopulmonary diseases. The specific anesthesia method was determined by the anesthesiologist.

### Operative techniques

The Lichtenstein and anterior Kugel mesh repair were performed as previously described in studies [12–14]. It should be emphasized that try not to open the peritoneal sac during surgery. If the hernia sac was opened inevitably, or it was large and the parts of it were tightly adhesive with the spermatic cord, it needed to be transected at the internal ring level, and the open hernia sac needed to be closed tightly with a running suture with 3–0 prolene. Drains were not routinely used.

### Postoperative management and follow-up

According to the basic principle of enhanced recovery after surgery (ERAS), postoperative analgesia was routinely performed for each patient, and the patient’s diet was restored as early as possible to control the amount of intravenous input. In addition, total fluid intake also needs to be controlled as appropriate. CAPD patients with incarcerated hernias who underwent emergency surgery for strangulation needed to receive postoperative temporary HD and resumed the preoperative CAPD regimen within 2–4 weeks. However, dialysis in most of the patients who underwent elective mesh hernioplasty was resumed postoperatively on days 1–3 with low-exchange volumes (1.0–1.5 L) and high-frequency exchanges (5–6 exchanges per day) for 2 weeks, which was designed by nephrologists depending on RRF. All patients gradually resumed to the original CAPD regimens within 4 weeks after the initiation of postoperative dialysis. The patients’ median duration of follow-up was 34 months (3–62 months). The demographic, perioperative, postoperative and dialysis data were recorded.

### Statistical analysis

Medians were used to describe continuous variables. Data were analyzed using SPSS 24.0 software version (SPSS Inc, Chicago, IL, USA).

## Results

The median age of the CAPD patients, who were all male with a median BMI of 23.1 kg/m^2^ (17.0–31.5 kg/m^2^), was 65 years (46–82 years). The characteristics of the patients are listed in Table 1. All hernias were primary hernias, and the median duration of CAPD before hernia formation was 16 months (2–61 months). The causes of renal failure were glomerulonephritis (n = 5), diabetes nephropathy (n = 7), hypertensive nephropathy (n = 2), polycystic kidney disease (n = 1), and others (n = 3). All patients have preoperative comorbidities, and 7 patients (39%) have more than three comorbidities.

**Table 1:** Demographic data

Demographic data	n
Patients/hernias	18/20
Age (years)	65 (46–82)
Gender(male/female)	18/0
BMI (kg/m^2^)	23.1 (17.0–31.5)
Comorbidities, n (%)	
Hypertension	12
Diabetes mellitus	9
Heart disease	7
Pulmonary disease	3
Liver disease	2
Duration of CAPD before hernia formation (months)	16 (2–61)
Etiology of renal failure, n (%)	
Glomerulonephritis	5
Diabetes nephropathy	7
Hypertensive nephropathy	2
Polycystic kidney disease	1
Other^a^	3

*BMI* body mass index

^a^1 case was renal artery stenosis and remaining 2 were unknown

According to the European Hernia Society (EHS) classification, the Lateral II type (n = 9) was the most common, followed by Lateral I (n = 6), Lateral III (n = 3), Medial I (n = 1) and the Medial II (n = 1). Most procedures (n = 12) were performed under general anesthetic, 2 were performed with a spinal anesthetic, and 4 procedures used local anesthesia due to severe cardiopulmonary disease. Except for 3 patients with incarcerated hernias who underwent emergency surgery, all patients underwent elective surgery following adequate perioperative preparation. Seventeen hernias were repaired with Lichtenstein techniques, which made up most of the tension-free repair surgeries. Bilateral inguinal hernias were simultaneously repaired using this approach, and the 3 remaining hernias were repaired with anterior Kugel techniques. Six hernia sacs (30%) were inevitably opened or transected during the surgery and were closed tightly with a running suture. The median operation time was 62.5 min (40–115 min) and median postoperative hospital stay for all patients was 3 days (1–7 day). The median operation times and median postoperative hospital stay did not differ significantly between the Lichtenstein group and anterior Kugel group (Table 2).

**Table 2:** Perioperative data

Perioperative data	n
Unilateral hernias, n (%)	
Left	5 (27.8%)
Right	11 (61.1%)
Bilateral hernias	2 (11.1%)
Lateral^a^, n (%)	
I	6 (30.0%)
II	9 (45.0%)
III	3 (15.0%)
Medial^a^, n (%)	
I	1 (5.0%)
II	1 (5.0%)
Mode of anaesthesia, n (%)	
General anaesthesia	12 (66.7%)
Spinal anaesthesia	2 (11.1%)
Local anaesthesia	4 (22.2%)
Operation, n (%)	
Elective	15 (83.3%)
Emergency	3 (16.7%)
Surgical approach, n (%)	
Lichtenstein procedure	17 (85.0%)
Anterior kugel procedure	3 (15.0%)
Operative time (min)	62.5 (40–115)
Lichtenstein group	60 (40–115)
Anterior Kugel group	65 (60–110)
Dissection or opening of hernia sac, n (%)	6 (30.0%)
Lichtenstein group	5 (25.0%)
Anterior Kugel group	1 (5.0%)
Length of hospital stay (days)	4 (1–7)
Lichtenstein group	3 (1–7)
Anterior Kugel group	3 (1–4)

^a^European Hernia Society classification

Postoperative complications included seroma (n = 2) and hematoma (n = 1), which were moderate and needed no additional medication. There were no wound or mesh infections, and no chronic pain of the groin area occurred. Except for 3 patients with incarcerated hernias, all patients received low-exchange volumes and high-frequency exchanges from 1 to 3 days after surgery for 2 weeks with gradual resumption of the original CAPD regimen within 4 weeks. Although two patients developed bacterial peritonitis 2 and 3 months after hernioplasty, both were cured after intraperitoneal antibiotic treatment without removal of the peritoneal catheter or interruption of CAPD. Moreover, no hernia recurrence or leakage of dialysate were found during the follow-up (Table 3).

**Table 3:** Postoperative complications

Complications	n
Seroma	2
Haematoma	1
Wound infection	0
Mesh infection	0
Recurrence	0
Chronic pain of groin area	0
Leakage of dialysate	0
Peritonitis^a^	2
Return to operation	0

^a^Both were bacterial peritonitis

## Discussion

**Fig. 1:**
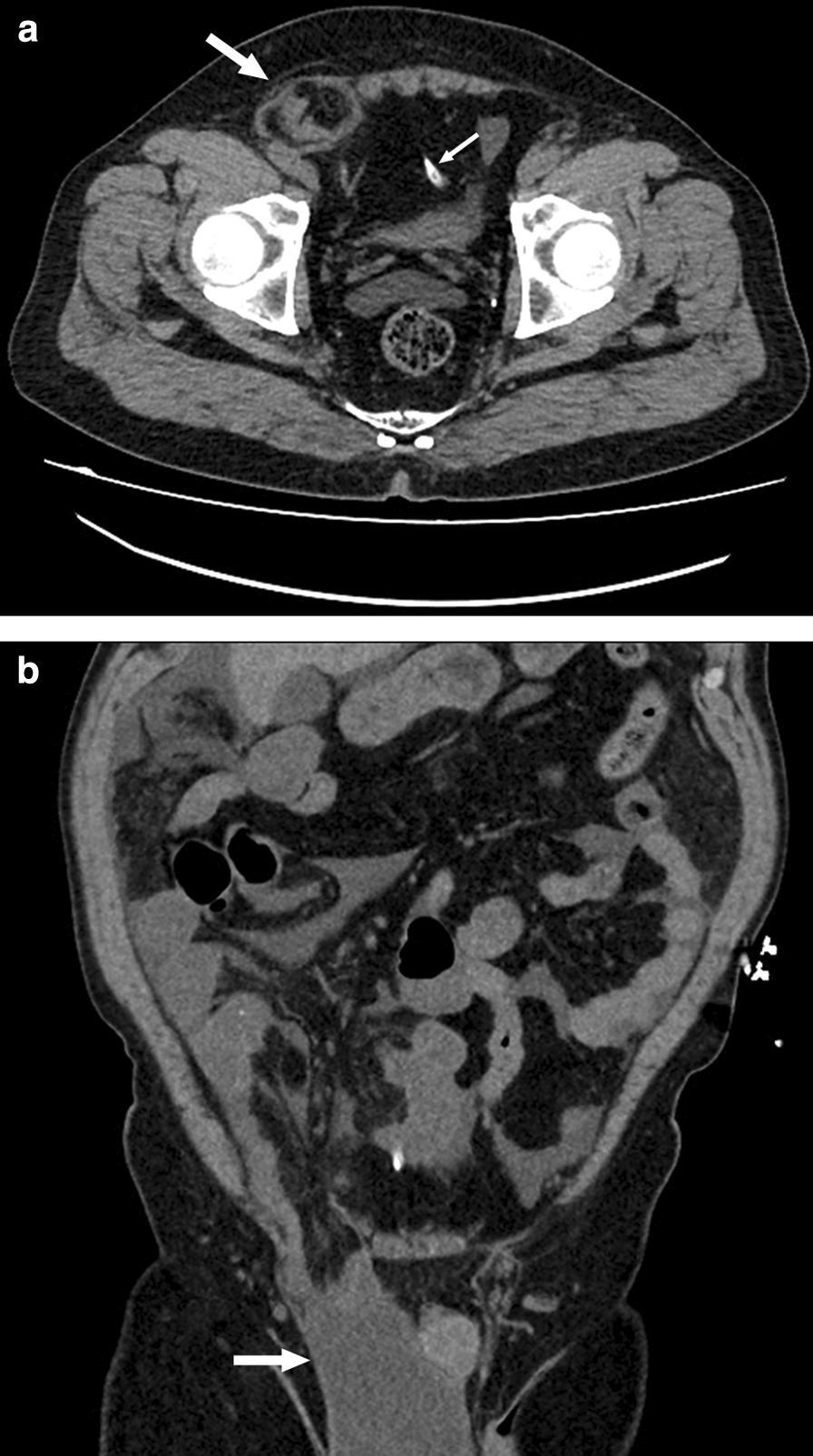
Nonenhanced CT images of patients with CAPD and hernia. **a** Axial CT image show a right inguinal hernia (thick arrow) and a CAPD catheter (thin arrow). **b** Coronal CT image show right inguinal hernia with dialysate (arrow)

Inguinal hernias in CAPD patients may cause local groin pain, swelling of the groin or genitals, ultrafiltration failure, and even bowel strangulation; however, similar to common hernia symptoms, most hernias in CAPD patients are asymptomatic, which might make them difficult to discover by nephrologists. Current modalities commonly used in studies to identify hernias in these patients include ultrasound, CT, peritoneal scintigraphy and CT peritoneography, and the latter modality is considered the reference standard for diagnosis [6, 7, 15]. Although it has a high detection rate and widespread availability, this modality has limitations; the process requires a strictly sterile technique, and patients need to be exposed to ionizing radiation [7]. Approximately 72% of hernias in this study were diagnosed by ultrasound, which requires an experienced sonographer to perform. Nonenhanced CT can also have good diagnostic value and can be an advantageous supplement for diagnosis (Fig. 1).

To reduce patient discomfort, increase ultrafiltration efficiency and reduce further damage caused by inguinal hernia, timely repair of the hernia in this unique population is advisable [8, 16]. The number of hernias repaired emergently may increase, often combined with bowel resection, whilst awaiting or refusing elective surgery, and mortality and complication rates may also increase [17, 18]. In addition, the patient on CAPD per se is adverse to recovery from inguinal herniorrhaphy. When this circumstance occurs, management is trickier and more difficult. Although, three patients underwent emergency surgery with Lichtenstein procedure for incarcerated hernias and recover well in the study, one patient in the same period had intestinal necrosis due to an incarceration time of more than 24 h; approximately 15 cm of the small intestine was intraoperatively removed, and simple repair with high ligation of the sac was performed and recurrence was found 2 months after surgery. Besides, the median postoperative hospital stay of emergency surgery was 5 days, which was longer than that of elective surgery.

In this study, we found no recurrences after tension-free mesh repair for inguinal hernia, including those patients who had early resumption of CAPD after repair, which demonstrates that tension-free mesh hernioplasty for CAPD patients is safe and feasible, which is consistent with the findings of other studies [5, 6, 9, 10]. Luk et al. [6] demonstrated that the Lichtenstein mesh repair remains the gold standard for patients with CAPD. Gianetta et al. [10] also claimed that the results of the Lichtenstein technique under local anesthesia for these high-risk subgroups are satisfactory. After verification with more patients in this study, we agree with the conclusion of the above studies. Furthermore, considering the unfavorable effect of uremia on wound healing, patients on CAPD are especially suitable for a minimally invasive procedure, so the Lichtenstein mesh repair should be the first choice. In addition, three patients who underwent the anterior Kugel procedure also achieved satisfactory results in this study. To the best of our knowledge, there is no report in the literature on the application of this surgical technique in the treatment of CAPD patients with inguinal hernia, and this is the first study.

The anterior Kugel procedure takes an anterior approach for preperitoneal repair and is a modified Kugel procedure. The procedure maintains most of the benefits of the standard Kugel procedure, such as strengthening all the defect areas of the myopectineal orifice simultaneously [19, 20]; moreover, the Kugel mesh can be used in limited and closed spaces via fixation by hydrostatic tissue pressure and requires no additional sutures [20], and the device is inserted into the preperitoneal space, which is contains no nerves or vessels, to avoid nerve and vessel injury. In addition, the technique is suitable for various types of inguinal hernias [20]. This provides a definite theoretical basis for the application of the procedure in CAPD patients. Certainly, application of this technique to repair inguinal hernias requires more familiarity with the inguinal anatomy, and the procedure is more complicated than the Lichtenstein procedure. However, the procedure is also minimally invasive and could be carried out using local anesthesia, which is of great significance to ESRL patients because some of them often have coexisting serious cardiopulmonary diseases.

A prospective randomized study reported that the recurrence rate between the Kugel and Lichtenstein procedures was not significantly different [21]. The Kugel procedure could eliminate “false” recurrences due to the lack of exploration of a missed hernia and provide the whole myopectineal orifice enhancement. As long as the surgeon is experienced, recurrence should be relatively rare in theory. Based on these circumstances, the Kugel technique is at least as safe as the Lichtenstein procedure. In addition, the procedure is suitable for CAPD patients with femoral hernia, which is difficult to address in the Lichtenstein procedure. No patients who underwent the anterior Kugel procedure experienced recurrence during the follow-up period in this study. In addition, the experience of anterior Kugel herniorrhaphy may be beneficial for laparoscopic hernia repair because both have the same operating space. It has already been reported that laparoscopic mesh repair of bilateral obturator hernias in CAPD patients may be considered a feasible operative approach [22], which provides a new way of thinking for the treatment of CAPD patients with bilateral inguinal hernia in good condition in the future.

There is no consensus on whether it is necessary to convert to HD or on the time needed to resume CAPD after surgery [4, 6, 9, 10, 16, 23]. One investigation revealed that some centers in the UK received temporary HD postoperatively and that the median duration of resuming CAPD was 4 weeks (1 day–8 weeks) [23]. In contrast, some studies advocated the conversion to intermittent PD for 2–4 weeks after surgery before restoring the preoperative CAPD regimen [6, 24]. Other studies have promoted postoperative PD schemes in which patients receive low-volume and high-frequency exchanges with a gradual regain of the preoperative CAPD regimen in 2–4 weeks [9, 16]. The last protocol was similar to that of our patients who received low-exchange volumes (1.0–1.5 L) and high-frequency exchanges (5–6 exchanges per day) from 1–3 days after surgery for 2 weeks with gradual resumption to the original CAPD regimens within 4 weeks. The early recurrence and dialysis leakage predicted by the scholars who advocated the conversion to HD did not occur. This has profound significance for these patients because it avoids the pain and risk of establishing and maintaining vascular access, reduces the consumption of medical resources and shortens the hospital stay. We consider that CAPD might be recovered early after surgery without immediate adverse effects on tension-free mesh repair for inguinal hernias.

Patients with ESRL often have poor nutrition, poor immune function, and poor wound-healing ability. In addition, due to the frequent infusion of peritoneal dialysis and the presence of prostheses as foreign bodies, infectious complications are likely to occur, particularly if the sterile technique needed for hernioplasty is not strict enough. We did not observe any wound or mesh infection in our group of patients after treatment with antibiotic prophylaxis, which was consistent with the findings of other studies [6, 9]. Although there were two patients in our study who developed bacterial peritonitis 2 and 3 months after hernioplasty, considering the time interval between surgery and infection, we believe that the infection was not related to surgery, as observed in the other studies [6, 10]; moreover, both patients were cured after intraperitoneal antibiotic treatment without removal of the peritoneal catheter or interruption of CAPD.

Of note, all hernias occurred after the start of CAPD in this study, the reason may be that these high-risk patients who hernias were diagnosed prior to PD catheter placement are often asymptomatic and are reluctant to undergo herniorrhaphy, or even with mild to moderate symptoms, most of them often choose to forbearance because of severe preoperative comorbidities. Simultaneous herniorrhaphy and PD catheter insertion may be a safe and effective treatment regimen for those who pre-existing hernia before the commencement of CAPD [25, 26].

Although the number of patients in the study is limited, the findings verify that tension-free mesh repair is safe and feasible for inguinal hernias in CAPD patients and that only moderate complications occur. The Lichtenstein mesh repair should be the first choice and is also suitable for CAPD patients with severe cardiopulmonary diseases for which general or spinal anesthesia are contraindicated. Anterior Kugel repair may be considered an effective surgical approach if technically feasible. Bridging HD seems unnecessary except for emergency surgery for incarcerated hernias. Using the correct method to address the hernia sac intraoperatively and maintaining close cooperation with nephrologists during the perioperative period are also essential to ensure the success of surgery and resumption of CAPD after surgery.

## Data Availability

The datasets used and analysed during the current study are available from the corresponding author on reasonable request.

## Abbreviations

CAPD: Continuous ambulatory peritoneal dialysis
PD: Peritoneal dialysis
ESRL: End-stage renal failure
HD: Hemodialysis
RRF: Residual renal function
CT: Computed tomography
ERAS: Enhanced recovery after surgery
BMI: Body Mass Index
EHS: European Hernia Society
